# Serum Albumin Modifies the Prognostic Meaning of BNP in Acute Decompensated Heart Failure

**DOI:** 10.1002/clc.70445

**Published:** 2026-08-13

**Authors:** Muneera O. AlTaweel, Elbadri I. Abdelgadir, Lulwah Al Turki, Ramy I. Abulikailik, Fahad Alharbi, Khamess O. Khamees, Waleed Gado

**Affiliations:** ^1^ Department of Cardiac Sciences, King Abdulaziz Hospital (KAH) Ministry of National Guard–Health Affairs (MNGHA) Al‐Ahsa Saudi Arabia; ^2^ King Abdullah International Medical Research Center (KAIMRC) Al‐Ahsa Saudi Arabia; ^3^ King Abdulaziz Hospital (KAH) Ministry of National Guard–Health Affairs (MNGHA) Al‐Ahsa Saudi Arabia; ^4^ College of Applied Medical Sciences King Saud bin Abdulaziz University for Health Sciences (KSAU‐HS) Al‐Ahsa Saudi Arabia; ^5^ Intensive Care Unit, King Abdulaziz Hospital (KAH) Ministry of National Guard–Health Affairs (MNGHA) Al‐Ahsa Saudi Arabia

**Keywords:** acute decompensated heart failure, B‐type natriuretic peptide, cardiorenal syndrome, effect modification, interaction, length of stay, risk stratification, serum albumin

## Abstract

**Background:**

BNP is widely used for risk stratification in acute decompensated heart failure (ADHF), but its prognostic meaning may differ across clinical phenotypes, particularly cardiorenal syndrome type 1 (CRS1).

**Hypothesis:**

We hypothesized that serum albumin modifies the association between BNP and adverse in‐hospital course in ADHF.

**Methods:**

In this retrospective cohort study, adults hospitalized with ADHF were classified as CRS1 or control. The primary endpoint was adverse in‐hospital course, defined as in‐hospital death and/or prolonged length of stay (≥ 75th percentile). BNP was analyzed on the natural log scale. Multivariable logistic regression included albumin, ln(BNP), and an albumin × ln(BNP) interaction term, adjusted for age, sex, systolic blood pressure, eGFR, ejection fraction, CRS status, body mass index, and diabetes mellitus.

**Results:**

In the complete‐case cohort (*N* = 292; events = 77), albumin significantly modified the BNP‐risk association (interaction *β* = −0.236 per 5 g/L; Wald *p* = 0.045; likelihood‐ratio test *p* = 0.050). The adjusted odds ratio for a 1‐unit increase in ln(BNP) was 1.81 at albumin 30 g/L, 1.42 at 35 g/L, and 1.12 at 40 g/L. Discrimination improved modestly with the interaction (AUC 0.714 vs. 0.726), and the high‐BNP/low‐albumin stratum showed the highest observed event risk.

**Conclusions:**

Serum albumin modifies the prognostic meaning of BNP in ADHF. Integrating albumin into BNP interpretation identifies a high‐risk “congestion plus vulnerability” phenotype and may support pragmatic triage and discharge planning in CRS‐enriched ADHF populations.

## Key message

In ADHF, low albumin levels amplify the BNP‐associated risk of an adverse in‐hospital course, enabling a simple two‐test phenotype to guide care intensity and discharge planning.

## Graphical abstract

Population: 293 patients hospitalized with acute decompensated heart failure (ADHF); BNP and serum albumin measured at admission.

Question: Does serum albumin change how BNP predicts an adverse in‐hospital course?

Method: Multivariable logistic regression with an albumin × ln(BNP) interaction term, adjusted for age, sex, systolic blood pressure, eGFR, ejection fraction, CRS1 status, BMI, and diabetes mellitus.

Key finding: Albumin significantly modified the BNP–risk relationship (interaction OR 0.79, 95% CI 0.62–1.00, *p* = 0.045). BNP's association with adverse outcomes was strongest in patients with low albumin and weakened as albumin rose.

Risk stratification (2 × 2): Adverse in‐hospital event rates ranged from 18.2% (normal albumin, low BNP) to 38.1% (low albumin, high BNP)—more than double the risk in the combined “congestion plus vulnerability” phenotype.

Take‐home message: BNP should not be interpreted in isolation in ADHF. Pairing it with serum albumin—a routine, inexpensive test—identifies a high‐risk subgroup that may warrant closer monitoring and more cautious discharge planning.

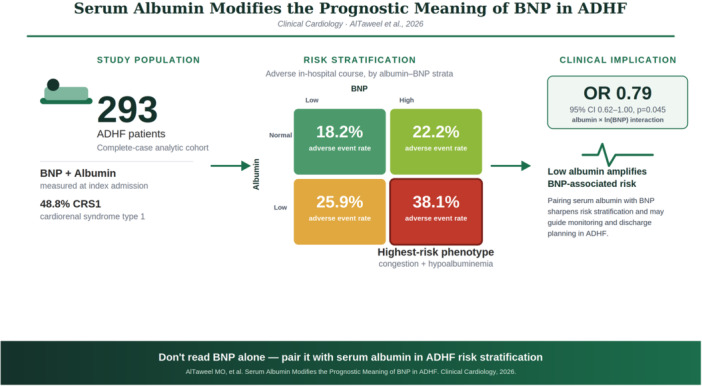

## 1 Introduction

Acute decompensated heart failure (ADHF) remains a leading cause of emergency presentations and hospitalizations, with substantial short‐term mortality and prolonged length of stay (LOS) despite advances in guideline‐directed therapy. Therefore, risk stratification during the index admission is central to timely escalation of care and appropriate discharge planning.

B‐type natriuretic peptide (BNP) is a cornerstone biomarker in ADHF for diagnosis and prognostication, reflecting myocardial wall stress and the burden of congestion. Its clinical utility is well established in landmark studies and contemporary guidelines [1, 2, 3, 4].

However, BNP values are influenced by clinical context, including renal dysfunction and the systemic consequences of congestion. Cardiorenal syndrome type 1 (CRS1), an acute deterioration in cardiac function that results in acute kidney injury (AKI), is common in ADHF and is associated with worse in‐hospital outcomes [5, 6].

Serum albumin is an inexpensive, widely available marker that reflects nutritional reserve, inflammation, hepatic congestion, hemodilution, and capillary leak. In heart failure populations, hypoalbuminemia has consistently been associated with higher mortality and rehospitalization risk and may reflect a vulnerable phenotype in which congestion translates into disproportionate organ dysfunction [7, 8, 9].

Although BNP and albumin have established prognostic roles individually, whether albumin modifies the prognostic meaning of BNP in ADHF, including in cohorts with a substantial burden of CRS1, remains poorly characterized. CRS1 status is a clinically relevant covariate in this setting given its associations with both BNP and albumin, though its own effect modification of the albumin–BNP relationship was not the focus of this analysis. We therefore tested whether albumin modifies the association between BNP and adverse in‐hospital course in ADHF, and whether this interaction can be translated into a pragmatic bedside risk‐stratification frameworK.

## 2 Methods

We conducted a retrospective observational cohort study of adults admitted with ADHF. Patients were classified as having ADHF with CRS1 or ADHF without CRS1 (control) using prespecified criteria: CRS1 was defined as ADHF precipitating AKI according to KDIGO criteria (serum creatinine rise ≥ 0.3 mg/dL [≥26.5 μmol/L] within 48 h, or ≥×1.5 baseline within 7 days, or urine output < 0.5 mL/kg/h for ≥6 h), consistent with the characterization of this parent cohort reported previously. CRS1 status was included as a covariate to account for its associations with both BNP and albumin; it was not evaluated as an effect modifier via interaction or stratified analysis in this study. The index time point was the ADHF admission (time zero), and follow‐up extended through hospital discharge or in‐hospital death. The primary objective was to evaluate whether serum albumin modifies the association between BNP and an adverse in‐hospital course.

Albumin and BNP were obtained from the index hospitalization, using the first available measurement recorded during admission for each patient; BNP was analyzed on the natural logarithmic scale due to right skewness. The primary endpoint was adverse in‐hospital course, defined as a composite of in‐hospital death and/or prolonged LOS, with prolonged LOS defined as LOS at or above the 75th percentile in the cohort. Secondary endpoints included prolonged LOS and in‐hospital death as individual components.

**Figure 1:**
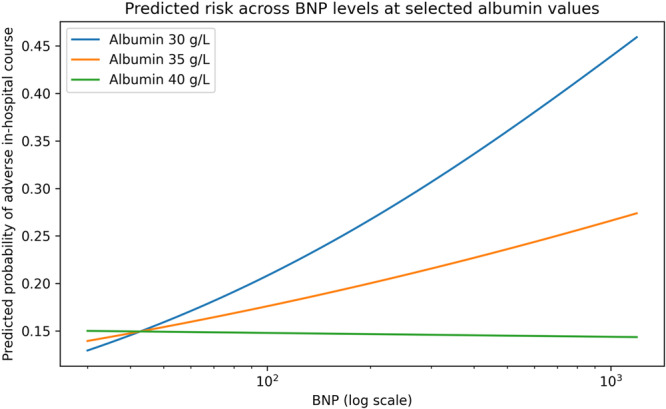
Predicted probability of an adverse in‐hospital course across BNP levels at selected albumin values. Predictions are derived from the fitted multivariable logistic regression model, with other covariates held at typical cohort values.

Baseline characteristics were summarized as mean (standard deviation) or median (interquartile range) for continuous variables and as counts (percentages) for categorical variables. For the primary analysis, multivariable logistic regression was used to model the primary endpoint, with albumin, log‐transformed BNP, and their interaction term (albumin × ln[BNP]) as key predictors. To aid clinical interpretation of effect modification, marginal effects and predicted probabilities were examined across BNP levels at clinically relevant albumin values (Figure 1). A parsimonious, clinically prespecified adjustment set was applied to reduce overfitting and preserve interpretability, including age, sex, systolic blood pressure, estimated glomerular filtration rate, left ventricular ejection fraction (as continuous EF% and/or phenotype categories), CRS status, body mass index (BMI), and diabetes mellitus (DM). BMI and DM were added to the adjustment set given their established associations with both BNP and albumin and their sufficient prevalence in this cohort (DM: 86.0% in CRS1, 80.7% in controls; BMI available in 292/293 patients). Additional variables associated with albumin and BNP in prior literature, including atrial fibrillation, chronic liver disease, nephrotic syndrome, active malignancy, and inflammatory states, were considered but not systematically captured in sufficient detail to permit reliable adjustment in this retrospectively assembled dataset; this is noted as a limitation. Results are reported as adjusted odds ratios with 95% confidence intervals. Analyses used complete‐case data for the variables included in each model, and missingness for key variables was reported. Given the low proportion of missing data (6.7% of eligible patients), a complete‐case approach was used. Because excluded patients had a higher prevalence of CRS1 and higher presenting systolic blood pressure, missingness may not have been entirely random, and this potential source of selection bias is acknowledged as a study limitation. Because only 6.7% of eligible patients were excluded and the principal biomarker variables (BNP, albumin, eGFR, and EF%) were similar between included and excluded patients, we elected to use a complete‐case approach rather than multiple imputation. All tests were two‐sided, with significance set at *p* < 0.05.

### 2.1 Ethics Statement

The study protocol was approved by the institutional review board (approval No. NRA26/006/2) and was conducted in accordance with applicable institutional and national ethical standards. The requirement for informed consent was waived due to the retrospective design and the use of de‐identified data.

## 3 Results

**Figure 2:**
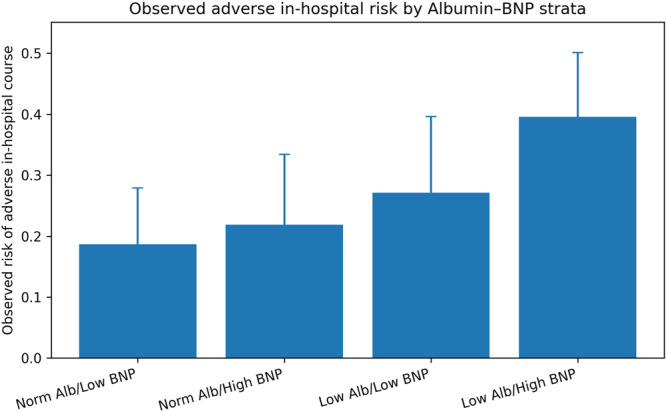
Observed risk of adverse in‐hospital course by albumin–BNP strata (2 × 2). Bars show the observed risk, and error bars show 95% Wilson confidence intervals.

Of 314 eligible patients, 21 (6.7%) were excluded because of missing data, yielding a complete‐case cohort of 293 patients. Compared with included patients, excluded patients had a higher prevalence of CRS1 (85.7% vs. 48.8%; *p* = 0.002) and higher systolic blood pressure at presentation (145 [124–155] vs. 125 [110–142] mmHg; *p* = 0.004), whereas age, ejection fraction, BNP, serum albumin, and eGFR were similar between groups (Supporting Information S1: Table 1). Among 293 patients in the complete‐case cohort, 143 (48.8%) met criteria for CRS1. In the primary multivariable model, additionally adjusted for BMI and diabetes mellitus (complete‐case *N* = 292, events = 77), serum albumin significantly modified the association between BNP and adverse in‐hospital course (albumin × ln[BNP] interaction *β* = −0.236 per 5 g/L; Wald *p* = 0.045; likelihood‐ratio test for interaction *p* = 0.050; Table 2). Clinically, the adjusted effect of BNP on risk was strongest at lower albumin and attenuated with higher albumin: the adjusted odds ratio (OR) for a 1‐unit increase in ln(BNP) (≈2.7‐fold higher BNP) was 1.81 at albumin 30 g/L, 1.42 at albumin 35 g/L, and 1.12 at albumin 40 g/L (Figure 1). Risk stratification using a simple 2 × 2 framework (albumin < 35 g/L and BNP ≥ cohort median) showed the highest adverse event rates in the high‐BNP/low‐albumin stratum (Table 1; Figure 2). Model discrimination improved modestly with the interaction (AUC 0.714 without interaction vs. 0.726 with interaction; ΔAUC 0.012). BMI was independently associated with adverse in‐hospital course (adjusted OR 1.04, 95% CI 1.00–1.09, *p* = 0.032); diabetes mellitus was not (adjusted OR 1.47, 95% CI 0.61–3.58, *p* = 0.394). The inclusion of BMI and diabetes mellitus resulted in only modest attenuation of the interaction term compared with the original model, with preservation of the direction and clinical interpretation of the effect.

**Table 1:** Baseline characteristics and outcomes, overall and by albumin–BNP strata (2 × 2).

Characteristic	Overall (*N* = 293)	Normal Alb (≥ 35 g/L) Low BNP (*n* = 88)	Normal Alb (≥ 35 g/L) High BNP (*n* = 63)	Low Alb (< 35 g/L) Low BNP (*n* = 58)	Low Alb (< 35 g/L) High BNP (*n* = 84)
Age, mean (SD)	68.0 (12.6)	67.3 (10.5)	68.4 (13.6)	68.3 (11.8)	68.3 (14.3)
Male, *n* (%)	155 (52.9)	38 (43.2)	43 (68.3)	26 (44.8)	48 (57.1)
CRS1, *n* (%)	143 (48.8)	30 (34.1)	37 (58.7)	27 (46.6)	49 (58.3)
SBP, mean (SD), mmHg	125.5 (21.1)	135.1 (19.5)	120.8 (20.1)	125.9 (22.2)	118.8 (19.2)
eGFR, median (IQR), mL/min/1.73 m^2^	44.0 (31.0–69.0)	62.0 (39.8–85.5)	41.0 (32.0–58.0)	48.5 (33.2–73.5)	33.0 (22.8–45.2)
EF%, mean (SD)	39.5 (14.9)	45.8 (13.0)	32.9 (14.8)	44.1 (14.8)	34.7 (13.3)
Albumin, mean (SD), g/L	34.1 (5.2)	38.3 (2.9)	37.6 (2.3)	30.6 (3.7)	29.5 (4.0)
BNP, median (IQR), pg/mL	246.0 (100.0–538.0)	81.5 (54.2–144.5)	530.0 (339.5–702.0)	112.0 (56.8–158.0)	546.5 (356.2–832.2)
LOS, median (IQR), days	5.0 (3.0–7.0)	4.0 (3.0–6.0)	5.5 (3.0–7.0)	5.0 (4.0–7.0)	6.0 (4.0–8.0)
Adverse in‐hospital course, *n* (%)	77 (26.3)	16 (18.2)	14 (22.2)	15 (25.9)	32 (38.1)
Prolonged LOS (≥p75), *n* (%)	66 (23.2)	15 (17.9)	13 (21.0)	12 (21.1)	26 (32.1)
In‐hospital death, *n* (%)	23 (8.0)	1 (1.1)	4 (6.5)	4 (7.0)	14 (17.3)

*Note:* Complete‐case cohort, *N* = 293. Low albumin is defined as < 35 g/L. High BNP is defined as BNP ≥ cohort median (246.0 pg/mL). Values are reported as mean (SD), median (IQR), or *n* (%), as appropriate.

In prespecified secondary analyses evaluating the individual components of the composite endpoint, the albumin × ln(BNP) interaction was not statistically significant for in‐hospital death alone (OR 0.91, 95% CI 0.60–1.39; *p* = 0.667; *n* = 288, 23 events) or for prolonged LOS alone (OR 0.83, 95% CI 0.67–1.04; *p* = 0.107; *n* = 284, 66 events). The primary outcome of this study remained the prespecified composite endpoint, which was selected a priori to improve statistical efficiency given the relatively low frequency of individual events, particularly in‐hospital mortality.

## 4 Discussion

This analysis shows that serum albumin modifies the prognostic meaning of BNP in ADHF. Rather than serving as independent additive risk markers, BNP and albumin interact, with BNP‐associated risk amplified in hypoalbuminemic patients and attenuated in those with higher albumin (Table 2; Figure 1). This effect modification defines a clinically actionable phenotype: a “congestion plus vulnerability/hemodilution” profile captured by two routine tests.

**Table 2:** Multivariable logistic regression for adverse in‐hospital course, including the albumin × ln(BNP) interaction and expanded adjustment set (BMI, diabetes mellitus).

Predictor	Adjusted OR	95% CI	*p* value
Albumin (per 5 g/L)	2.40	0.66–8.77	0.186
ln(BNP)	7.53	1.48–38.42	0.015
Albumin × ln(BNP) (per 5 g/L)	**0.79**	**0.62–1.00**	**0.045**
Age (years)	1.01	0.98–1.04	0.487
Male sex	1.24	0.66–2.35	0.508
Systolic BP (mmHg)	0.99	0.97–1.00	0.058
eGFR (mL/min/1.73 m^2^)	1.00	0.99–1.02	0.986
Ejection fraction (%)	1.03	1.01–1.06	0.019
CRS1 (vs. control)	1.29	0.63–2.61	0.487
BMI (kg/m^2^)	1.04	1.00–1.09	0.032
Diabetes mellitus	1.47	0.61–3.58	0.394

*Note:* Adjusted odds ratios (OR) with 95% confidence intervals are reported; albumin is scaled per 5 g/L. *N* = 292. The bolded row is the primary interaction term of interest.

BNP reflects myocardial wall stress and congestion and is endorsed by major guidelines for diagnostic and prognostic use in ADHF [1, 2, 3, 4]. Albumin integrates multiple pathways associated with ADHF severity, including malnutrition, inflammation, hepatic congestion, and reduced oncotic pressure, all of which may exacerbate edema and impair organ perfusion [7, 8, 9].

Our findings align with prior work showing that hypoalbuminemia is associated with worse short‐ and long‐term outcomes in heart failure, including in cohorts with acute decompensation [8, 10]. The novel contribution here is demonstrating that albumin is not merely a parallel risk marker but an effect modifier that alters the BNP–risk gradient, enabling more nuanced interpretation of natriuretic peptide values.

Clinically, this interaction supports the use of albumin to contextualize BNP levels when triaging ADHF patients, particularly in populations with a high burden of CRS1, where biomarker interpretation can be confounded by renal dysfunction [5, 6]. A simple bedside 2 × 2 classification (Table 1; Figure 2) identifies a high‐risk subgroup (high BNP/low albumin) that may warrant earlier intensive monitoring, more aggressive decongestion strategies, and closer discharge planning.

This study should be interpreted with consideration of its retrospective design and single‐cohort structure. Biomarker measurements were obtained during routine care, and unmeasured confounding may persist despite adjustment. Although we expanded the adjustment set to include BMI and diabetes mellitus based on their availability and prevalence in this cohort, other variables previously associated with albumin and BNP levels, including atrial fibrillation, chronic liver disease, nephrotic syndrome, active malignancy, and inflammatory states, were not systematically captured in sufficient detail to permit reliable adjustment in this retrospectively assembled dataset; residual confounding from these factors cannot be excluded. Although the proportion of excluded patients was small, excluded patients were more likely to have CRS1 and presented with higher systolic blood pressure. Consequently, the analytic cohort may modestly underestimate the prevalence of CRS1 in the underlying ADHF population, and some selection bias cannot be excluded. Importantly, BNP, serum albumin, eGFR, and ejection fraction were similar between included and excluded patients, mitigating concern that exclusions materially altered the biomarker relationships under study. The in‐hospital composite endpoint, while clinically pragmatic, may reflect heterogeneous mechanisms (death and prolonged LOS). Neither component of the composite reached statistical significance independently when modeled alone, most notably for in‐hospital death, which was rare (23/293 events) and yielded an imprecise estimate. With only 23 in‐hospital deaths, the mortality‐specific model had limited power to detect modest interaction effects; this is an expected consequence of evaluating low‐frequency events individually and is consistent with the rationale for using a composite endpoint in a cohort of this size. Prolonged LOS alone showed a similar direction of effect but did not reach significance, consistent with LOS being influenced by non‐cardiac and logistic factors beyond the exposures studied here. Finally, interaction effects can be sensitive to model specification. The interaction remained statistically significant, although attenuated, after additional adjustment for BMI and diabetes mellitus, suggesting that the observed effect modification is not fully explained by obesity‐ or diabetes‐related differences in BNP or albumin concentrations. We further translated the interaction into predicted probabilities for interpretability (Figure 1).

In conclusion, albumin significantly modified the association between BNP and adverse in‐hospital course in this cohort. Incorporating albumin into BNP interpretation improves clinical phenotyping and identifies a subgroup in whom BNP indicates markedly higher risk (Figures 1 and 2).

## Author Contributions

Muneera O. AlTaweel contributed to conceptualization, methodology, data curation, and writing (original draft and revision), and served as corresponding author. Elbadri I. Abdelgadir and Khamess O. Khamees contributed to data curation. Lulwah Al Turki and Ramy I. Abulikailik contributed to the definition and clinical characterization of cardiorenal syndrome type 1 used in this study. Fahad Alharbi provided intensive care unit‐based clinical input on acute severity assessment. Waleed Gado supervised the overall manuscript. All authors read and approved the final manuscript.

## Conflicts of Interest

The authors declare no conflicts of interest.

## Supporting information


Supporting File

## Data Availability

The data that support the findings of this study are available on request from the corresponding author. The data are not publicly available due to privacy or ethical restrictions.
