# Supplementary material for: Serum Albumin Modifies the Prognostic Meaning of BNP in Acute Decompensated Heart Failure
Source: Clin Cardiol. 2026 Aug 13;49(8):e70445. doi: 10.1002/clc.70445 (PMC13470876; doi:10.1002/clc.70445)
Supplement: Supplementary file 1 [file CLC-49-e70445-s001.docx]

**Supplementary Table S1**

Comparison of patients included versus excluded from the complete-case analysis. Of 314 eligible patients, 21 (6.7%) were excluded because of missing data in one or more model variables (age, sex, systolic blood pressure, eGFR, ejection fraction, CRS status, albumin, BNP, or outcome), yielding the complete-case cohort of 293. Continuous variables are reported as median (IQR) given non-normal distributions; comparisons used the Mann-Whitney U test. Categorical variables are reported as n (%); comparisons used the chi-square test.

| **Characteristic** | **Included (N=293)** | **Excluded (N=21)** | **p-value** |
| --- | --- | --- | --- |
| Age, years — median (IQR) | 68.0 (61.0–76.0) | 66.0 (59.2–69.8) | 0.189 |
| Male, n (%) | 155 (52.9) | 13 (61.9) | 0.567 |
| CRS1, n (%) | 143 (48.8) | 18 (85.7) | 0.002 |
| Systolic BP, mmHg — median (IQR) | 125.0 (110.0–142.0) | 145.0 (124.0–155.0) | 0.004 |
| eGFR, mL/min/1.73m² — median (IQR) | 44.0 (31.0–69.0) | 37.0 (28.0–45.2) | 0.106 |
| Ejection fraction, % — median (IQR) | 37.5 (25.0–55.0) | 52.5 (37.5–55.0) | 0.160 |
| Serum albumin, g/L — median (IQR) | 35.0 (31.0–37.0) | 38.0 (33.0–38.5) | 0.105 |
| BNP, pg/mL — median (IQR) | 246.0 (100.0–538.0) | 364.0 (105.7–984.0) | 0.354 |
